# Human Health Risk Evaluation of Organophosphate Insecticide Residues in Post-Harvest Cowpea in Gwagwalada, Abuja, Nigeria

**DOI:** 10.5696/2156-9614-10.28.201203

**Published:** 2020-11-19

**Authors:** Motunrayo G. Akande, Fatimah S. Sanni, Ndidi G. Enefe

**Affiliations:** 1 Department of Veterinary Pharmacology and Toxicology, Faculty of Veterinary Medicine, University of Abuja, Abuja, Nigeria; 2 Department of Veterinary Biochemistry and Physiology, Faculty of Veterinary Medicine, University of Abuja, Abuja, Nigeria

**Keywords:** organophosphates, malathion, parathion, ethion, carbophenothion, risk evaluation, cowpea

## Abstract

**Background.:**

Cowpea is a leguminous crop commonly grown and eaten in Nigeria. Organophosphate insecticides are frequently used to control insect populations in cowpea crops.

**Objectives.:**

The present study was conducted to investigate the concentrations of organophosphate insecticide residues in cowpea varieties in Gwagwalada, Nigeria, and assess health risks to consumers.

**Methods.:**

Samples of brown and white cowpea varieties were collected from Gwagwalada market, Abuja, Nigeria. Concentrations of organophosphate insecticide residues in the cowpea samples were analyzed by gas chromatography-mass spectrometry with selective ion monitoring. Risk evaluation was carried out by the determination of estimated daily intake, hazard quotient and chronic hazard index.

**Results.:**

The organophosphates detected in the cowpea varieties were malathion, parathion, ethion and carbophenothion. The concentrations of insecticides in the cowpea types were higher than the maximum residue limits recommended by the European Union (EU) and the Agency for Toxic Substances and Disease Registry (ATSDR). The hazard quotient values were less than 100% for malathion, parathion and ethion in the cowpea varieties for adults and children. The hazard quotient of carbophenothion for adults was below 100% for the cowpea types, while the hazard quotient surpassed 100% for children. The chronic hazard indexes for children were 364% and 276% for the brown and white cowpea types, respectively.

**Conclusions.:**

The results obtained in the present study indicate that consumers, particularly children, may be exposed to health risks through the consumption of cowpea types. Consequently, monitoring and regulation of organophosphate insecticide usage in Nigeria should be intensified.

## Introduction

Cowpea (*Vigna unguiculata*) is a legume that is regularly planted in the tropical and subtropical regions of the world for consumption by humans and animals.^1^ Nigeria, located in West Africa, is the world's principal producer and consumer of cowpea.^2^ Insects are mainly responsible for poor yields of cowpea grown in the African continent, with adverse effects on the vital parts and crucial phases of the crop.^3^ Thus, farmers in different parts of the world use pesticides on crops for the purpose of protecting them from insects, other pests and disease outbreaks.^4^

Pesticides are potentially poisonous to human beings and can elicit acute and chronic health effects based on the quantity and routes of exposure in people.^5^ Agriculturists and people who come into contact with pesticides during and immediately after their application are at a high risk of exposure, whereas the general public are exposed extensively to minor quantities of pesticides through food and water. 5

Therefore, it is important to monitor the levels of various pesticides, including organophosphates, in food samples in order to safeguard the wellbeing of consumers.

The present research was undertaken to assess the health risks associated with the exposure of adults and children to residues of organophosphates in brown and white cowpea varieties in Gwagwalada, Abuja, Nigeria.

## Methods

Gwagwalada market is the foremost market in Gwagwalada, Abuja, Nigeria. Abuja is the capital city of Nigeria and is located in its north-central region. Brown and white cowpea types can be purchased from Gwagwalada market, as well as other edible household staples. Traders from different parts of Nigeria sell cowpeas in the market.

### Collection of cowpea samples

The cowpea types (500 g each) were purchased through a convenience randomization method from diverse traders in the market for the evaluation of residues of organophosphate insecticides and human health risk assessment in August 2019.

### Extraction of cowpea samples

Extraction of the samples was performed in accordance with the method described by Anastassiades *et al.*^6^ The cowpea samples were ground to make a consistent sample representative of the product. A total of 15 g of the ground product was weighed into a clean 50 ml tube. Subsequently, 15 ml of 1% acetic acid in acetonitrile (vol./vol.) and an appropriate quantity of an internal standard solution was added to it. The grounded output was centrifuged for one minute at 1500 rpm to separate the solid material before extraction.

### Purification of extracts

Silica gel was activated by heating at 130^°^C for 16 hours and the activated silica gel was stored in a desiccator. Five (5) g of silica gel was packed in a glass column and 1 g of anhydrous sodium sulfate was added. The column was conditioned with 20 ml n-hexane, and the hexane was eluted into a beaker labelled “waste.” Two (2) ml extract was added to the top of the column. Then the sample vial was rinsed with additional hexane to complete quantitative transfer. Another 10 ml of n-hexane was added to the column and eluted to waste. Subsequently, 10 ml dichloromethane and hexane (1:1) was added and the eluent was collected before the column head dried out. The eluent was concentrated to approximately 2 ml for analysis. The supplementary solvent was evaporated to dryness under a stable stream of nitrogen.

**Abbreviations**
*ADI*: Acceptable daily intake
*ATSDR*: Agency for Toxic Substances and Disease Registry
*CHI*: Chronic hazard index
*EDI*: Estimated daily intake
*EUPD*: European Union Pesticides Database
*FAO*: Food and Agriculture Organization of the United Nations
*FIR*: Food ingestion rate
*HQ*: Hazard quotient
*WHO*: World Health Organization

### Organophosphate insecticide gas chromatography-mass spectrometry

The gas chromatography-mass spectrometer (GC-MS) was adjusted to perfluorotributylamine for the verification of m/z 69, 219, 502 and additional conditions of the GC-MS. The gas chromatography-mass spectrometer was operated in selective ion monitoring (SIM) and scan mode. An Agilent 7820A gas chromatograph (Agilent Technologies, California) with an electron-impact source coupled to an inert mass spectrometer was utilized. Helium was used as a carrier gas at 1.2 mL/min, a pressure of 0.71322 psi and an average velocity of 40.00 cm/sec. The injection temperature was 250°C, while the purge flow employed was 15.0 mL/min at 0.75 minute. The programming of the temperature of the oven was as follows: 60°C (1 minute), increased afterwards from 25°C/min to 210°C (1 minute), and then 20°C/min to 310°C (5 minutes). The run time lasted for 16 minutes.

### Determination of percentage recovery

Two samples of ground cowpea weighing 15 g each were selected. One sample was spiked with 10 mg/L standard mixture that contained the organophosphate insecticides of interest. Subsequently, the mixture was properly mixed together by a vortex mixer. The remaining sample was left as a control (unspiked). The samples were extracted, purified and evaluated as described in the previous sections. The recoveries of the organophosphate insecticides were verified by comparing the peak areas of the organophosphate insecticides after spiking with those that were unspiked (the control). The percentage recoveries were derived from the concentration of the analytes that were detected in the spiked samples.

### Evaluation of the limit of detection and limit of quantification

The gas chromatography-mass spectrometer was externally calibrated using five standards at 2, 5, 10, 100 ppm. Ten (10) 1 L aliquots of tap water were fortified with 0.0004 mg/L of organophosphate insecticide standard mix. The extracts were concentrated to 1 ml and were then analyzed by gas chromatography using the appropriate instrument parameters.

The limit of detection is 3 multiplied by the SD of the blank signal. It was computed as the concentration at which the baseline noise to signals ratio is 3 at the retention time for each organophosphate insecticide.^7–10^ The limit of quantification was the concentration at a signal-to-noise ratio of 10.^10^

### Human health risk assessment

The estimated daily intake (EDI) of the organophosphate insecticides (malathion, parathion, ethion and carbophenothion) was determined based on their mean concentration in each cowpea type and the daily intake in grams. The food supply value of cowpea was 18 kg/capita/year according to Food and Agriculture Organization (FAO), 2000.^11^ The food supply value was divided by the number of days in the year (365 days). The result obtained was an intake of 0.04932, approximately 0.049 kg/capita/day, which is the food ingestion rate (F_IR_) of cowpea in Nigeria. Equation 1 was used for the calculation of the EDI.^12,13^

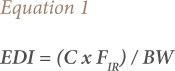
where, C is the dry weight concentration of the organophosphate insecticide in the cowpea variety in mg kg^−1^, F_IR_ is the daily F_IR_ in kg/day and BW is the reference body weight of 60 kg for an adult human. A reference body weight of 16 kg for children between one and six years old was used in this research.^12,14^

### Hazard quotient

The hazard quotient (HQ) was regarded as the probable risk of undesirable health effects from pesticide mixtures to specify the long-term assessment of risk and was computed by dividing the EDI by the pertinent acceptable daily intake (ADI) and multiplying by 100, Equation 2.^15,16^


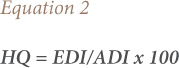


The ADI is defined as the quantity of a definite chemical that can be ingested daily for the life span of a human without substantial health risks.^17^

### Chronic hazard index

The HQ was estimated for the pesticides and cowpea types and the results were added up to derive a chronic hazard index (CHI), where CHI = ΣHQ.^15^ A CHI exceeding 100 indicates that the cowpea varieties pose a risk to consumers. However, a CHI lower than 100 indicates that the cowpea types are fit for consumption.^15,18^

### Data analysis

The results obtained in this study were stated as mean ± standard error of the mean. Data analysis was carried out with the Mann–Whitney two-tailed test. The Statistical Package for Social Sciences (version 23.0, New York) was deployed. Values of P<0.05 were considered statistically significant.

## Results

Linearity, limit of detection, limit of quantification and percentage recoveries of the four organophosphate insecticides (malathion, parathion, ethion and carbophenothion) are stated in Table 1. The limit of quantification was determined as follows:

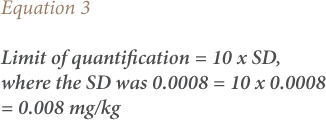


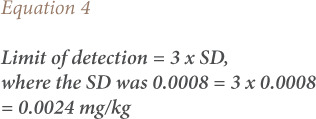



**Table 1:** Limit of Detection, Limit of Quantification and Percentage Recoveries of Organophosphate Insecticides Detected in Cowpea Samples

**Organophosphate insecticide**	**Linearity (R^2^)**	**Limit of detection (mg/kg)**	**Limit of quantification (mg/kg)**	**Recovery (%)**
Malathion	1.000	0.0024	0.008	79
Parathion	1.000	0.0024	0.008	99
Ethion	1.000	0.0024	0.008	90
Carbophenothion	1.000	0.0024	0.008	91

The results of the recovery tests ranged between 79% and 99% as stated in Table 1.

### Concentrations of organophosphate insecticides in cowpea samples

**Figure 1:**
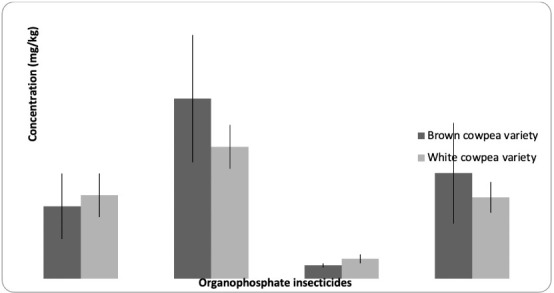
Mean concentrations of the organophosphate insecticides detected in the brown and white cowpea (Vignus unguiculata) varieties.

The mean concentrations of the four organophosphate insecticides (malathion, parathion, ethion and carbophenothion) detected in the cowpea varieties are depicted in Figure 1. Malathion, parathion, ethion and carbophenothion were detected in all the samples. In the brown cowpea, parathion had the highest mean value (0.82±0.29) mg/kg, while ethion had the lowest mean value (0.06±0.01) mg/kg. In the white variety of cowpea, parathion had the highest mean value (0.60±0.10) mg/kg, while ethion had the lowest mean value (0.09±0.02) mg/kg. There was no significant difference in the mean values of the investigated insecticides across cowpea types.

Maximum residue limits of the detected organophosphate insecticides in cowpea have not been reported by the FAO/World Health Organization (WHO) Codex Alimentarius, as shown in Table 2.^19^ However, the levels of malathion, parathion and ethion analyzed in this study surpassed the maximum residue limit set by the European Union Pesticides Database (EUPD) (for beans without pods) and the Agency for Toxic Substances and Disease Registry (ATSDR).^20,21^

**Table 2:** Comparison of Data Obtained in the Present Study with Acceptable Daily Intakes and Maximum Residue Limits

**Cowpea Variety**	**Malathion**	**Parathion**	**Ethion**	**Carbophenothion**
Brown	0.33± 0.15	0.82 ± 0.29	0.06±0.01	0.48±0.23
White	0.38 ± 0.10	0.60 ± 0.10	0.09±0.02	0.37± 0.07
FAO/WHO maximum residue limit (mg kg^−1^)^19^	2 (for dry beans)	Not reported	Not reported	Not reported
EUPD maximum residue limit (mg kg^−1^)^20^	0.02 (for beans without pods)	0.05 (for beans without pods)	0.01 (for beans without pods)	Not approved
ATSDR minimal risk limit (mg kg^−l^)^21^	0.02	0.009	0.0004	Not tolerated
UNEP/FAO/WHO ADI (mg kg^−1^)^22^	0.02	0.005	0.002	0.0005

Carbophenothion is not reported for use by the FAO/WHO, neither is it approved for use by the EUPD nor are its residues tolerated according to the ATSDR *(Table 2).*

### Human health risk assessment for adults and children

The EDI values for adults and children are presented in Table 3. The EDI for malathion, parathion and ethion in adults and children for both cowpea types were lower than the ADI for each organophosphate insecticide as reported by the joint United Nations Environment Programme (UNEP)/FAO/WHO.^22^ However, the EDI for carbophenothion in adults was lower than the ADI. The EDI exceeded the ADI for the cowpea types for children (*Table 3*).

**Table 3:** Estimated Daily Intake, Acceptable Daily Intake, Hazard Quotient and Hazard Index of Organophosphates Across Cowpea Types for Adults and Children

**Cowpea type**	**Age group**	**Organophosphate**	**EDI**	**ADI^22^**	**HQ (%)**	**HI**
Brown	Adults	Malathion	0.00027	0.02	1.35	2.9
		Parathion	0.00067	0.005	13.4	23.2
		Ethion	0.000049	0.002	2.45	6.15
		Carbophenothion	0.00039	0.0005	78	138
White	Adults	Malathion	0.00031	0.02	1.55	2.9
		Parathion	0.00049	0.005	9.8	23.2
		Ethion	0.000074	0.002	3.7	6.15
		Carbophenothion	0.00030	0.0005	60	138
Brown	Children	Malathion	0.0010	0.02	5	11
		Parathion	0.0025	0.005	50	86
		Ethion	0.00018	0.002	9	23
		Carbophenothion	0.0015	0.0005	300	520
White	Children	Malathion	0.0012	0.02	6	11
		Parathion	0.0018	0.005	36	86
		Ethion	0.00028	0.002	14	23
		Carbophenothion	0.0011	0.0005	220	520

Abbreviation: HI, hazard index.

### Hazard quotient

The outcomes obtained for the HQ in adults and children for the cowpea types are shown in Table 3. The HQs recorded for malathion, parathion and ethion were below 100% for the cowpea types for adults and children. In addition, the HQ for adults was below 100% for brown and white cowpeas for carbophenothion. The HQ for carbophenothion exceeded 100% for children in the brown and white cowpea types (300% and 220%, respectively), as seen in Table 3.

### Chronic hazard index

The HQs were summed up and the CHI for the organophosphate insecticides detected in this study is shown in Table 4. The CHI for the brown and white cowpea types for adults were 95.20% and 75.05%, respectively, and these values are less than 100%. On the other hand, for children, the CHI obtained for the brown and white cowpea types were 364% and 276%, respectively, and these values surpassed 100% and may indicate a possible health risk for children who consume cowpeas.

**Table 4:** Chronic hazard index of the organophosphate insecticide residues detected in the cowpea types

**Cowpea Type**	**Age group**	**Chronic Hazard Index (%)**
**Brown**	Adults	95.20
	Children	364
**White**	Adults	75.05
	Children	276

## Discussion

In the current study, four organophosphate insecticides (malathion, parathion, ethion and carbophenothion) were detected in all the brown and white varieties of cowpea analyzed. According to Olulakin *et al*., malathion and parathion are among the most commonly used organophosphate insecticides in Nigeria.^23^ Additionally, Jean *et al.*, detected malathion in cowpea samples in Cameroon (West Africa).^24^ These findings affirm our results and suggest that farmers in Nigeria might be applying additional organophosphate insecticides to cowpeas, including ethion and carbophenothion. It is believed that integrated pest management methods can help to solve the problem of pesticide residues in food by promoting the use of natural pest control mechanisms by the farmers, by ensuring the reduction of pests, and by maintaining the usage of pesticides by the farmers at levels that do not constitute risks to human beings, animals and the ecosystem in ways that are sustainable economically.^5^

The exposure of consumers to plant protection product residues (e.g. organophosphate insecticides) in foodstuffs is weighted in relation to the ADI and the ratio is an expression of the risk/safety for consumers when these products are used.^25^ The ADI refers to the quantity of a definite chemical that can be ingested daily for a life span without substantial health risks.^17^ In a situation where the definite exposure through pesticide residues is greater than the ADI, authorization should be prohibited for certain crops, longer pre-harvest periods must be computed, or approval must be denied.^25^

In the present investigation, the EDI for malathion, parathion and ethion in adults and children in both cowpea types were lower than the ADI for each organophosphate insecticide as reported by the joint UNEP/FAO/WHO.^22^ Additionally, the EDI for carbophenothion in adults was lower than the ADI. However, the EDI for carbophenothion exceeded the ADI for the cowpea types for children.

Carbophenothion is not approved for use by the FAO/WHO, European Union or the ATSDR. Its detection in the cowpea varieties in this investigation raises some concerns. Carbophenothion is an insecticide and acaricide.^26,27^ It is an organophosphate insecticide with protracted residual activity.^28^ The United States Environmental Protection Agency (USEPA) has classified carbophenothion as a Category I - highly toxic pesticide.^28^ It may boost the toxicity of malathion and other compounds, as well as their conversion into lethal substances in the body.^29^ The results of the present study suggest that the detection of carbophenothion in the cowpea types could be attributed to inappropriate pesticide use by farmers and could present a human health risk.

Furthermore, the maximum residue limit of the organophosphate insecticides identified in the cowpea types have not been reported by the FAO/WHO Codex Alimentarius, as depicted in Table 2.^19^ However, the levels of malathion, parathion and ethion analyzed in the present study exceeded the maximum residue limit reported by the EUPD (for beans without pods) and ATSDR.^20,21^ These findings may indicate some type of misuse or abuse of these chemicals in the Nigerian environment.

Moreover, the HQ values were greater than 100% for carbophenothion in the cowpea types for children. In addition, the CHI values were higher than 100% for children for the cowpea varieties and this may signify likely health risks. Children are more vulnerable to the detrimental effects of pollutants compared to adults due to their smaller body weights and dynamic developmental processes.^4,30^

There is a need for intensified monitoring and regulation of the residues of organophosphate insecticides in cowpea in Nigeria in order to preclude adverse health risks in the population, especially children.

## Conclusions

The present study provides information on the level of contamination of brown and white cowpea varieties with organophosphate insecticides and risk evaluation in adults and children in Gwagwalada, Abuja, Nigeria. The estimated daily intake for carbophenothion (an unauthorized and extremely toxic organophosphate insecticide) surpassed the acceptable daily intake for children in the cowpea samples. Children may be exposed to greater health risks from the consumption of the cowpea types based on the hazard quotient and chronic hazard index obtained in the present study.

The present study did not evaluate the potential variability in the levels of the organophosphate insecticides over time or the probable impacts of seasonal variations and further investigations are needed. In addition, the current study did not provide information on the farming context of the study.

It is recommended that the monitoring and regulation of organophosphate insecticide usage in Nigeria should be enhanced. In addition, effective educational programs, such as integrated pest management, should be organized for farmers to expand their knowledge of the appropriate use of pesticides in order to prevent health risks to the general population.
